# Supplementary material for: Pericyte-derived fibrotic scarring is conserved across diverse central nervous system lesions
Source: Nat Commun. 2021 Sep 17;12:5501. doi: 10.1038/s41467-021-25585-5 (PMC8448846; doi:10.1038/s41467-021-25585-5)
Supplement: Supplementary file 1 — Supplementary Information [file 41467_2021_25585_MOESM1_ESM.pdf]

SUPPLEMENTARY INFORMATION

Pericyte-derived fibrotic scarring is conserved across diverse central nervous system lesions

Authors: David O. Dias, Jannis Kalkitsas, Yildiz Kelahmetoglu, Cynthia P. Estrada, Jemal Tatarishvili, Daniel Holl, Linda Jansson, Shervin Banitalebi, Mahmood Amiry-Moghaddam, Aurélie Ernst, Hagen B. Huttner, Zaal Kokaia, Olle Lindvall, Lou Brundin, Jonas Frisén and Christian Göritz

CONTENTS:

Supplementary Figures (Pages 2-21)
Supplementary Tables (Pages 22-25)

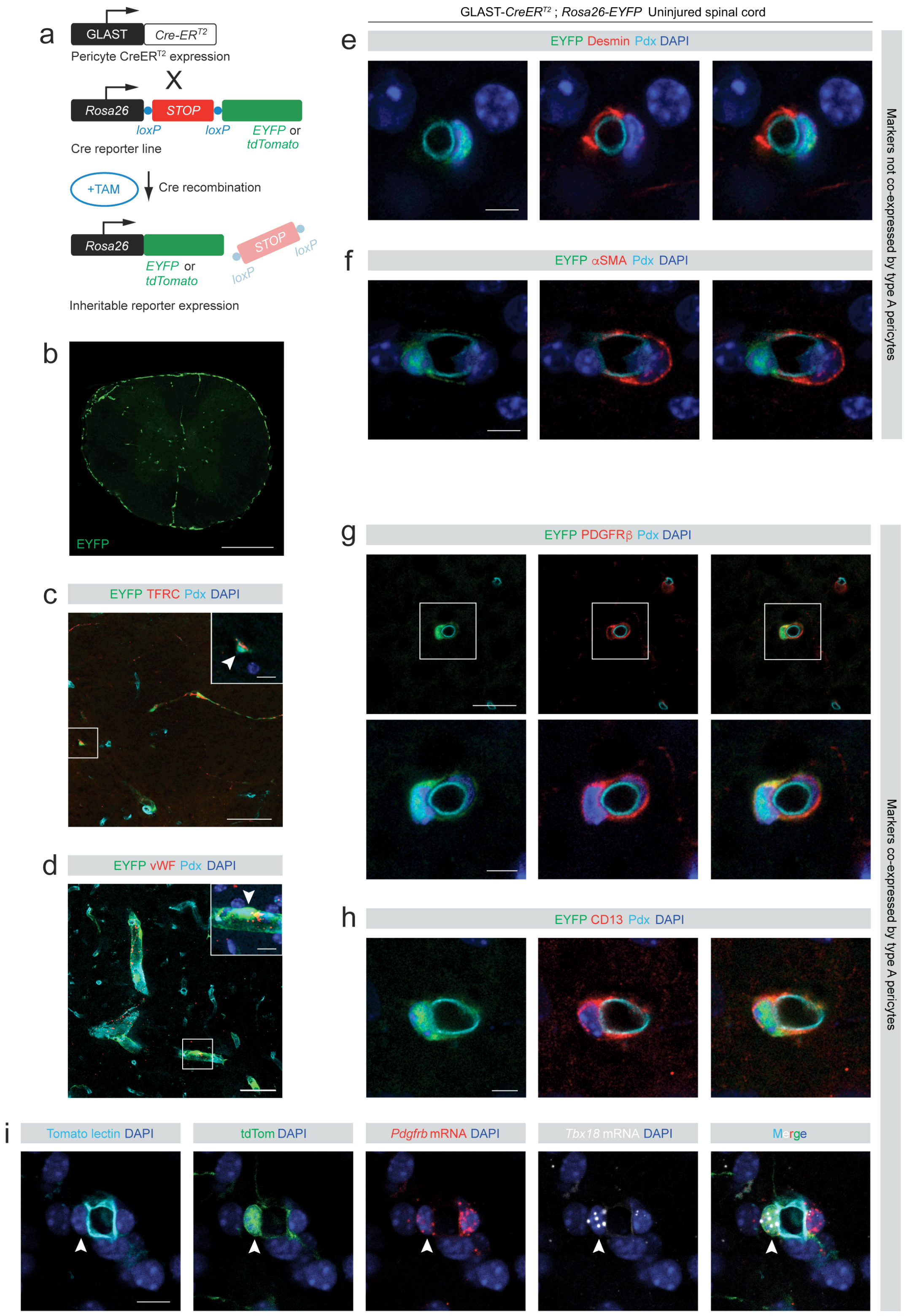

Supplementary Figure 1 | **Genetic labeling of type A pericytes in the adult spinal cord**

(**a**) Schematic depiction of the strategy to label a subset of perivascular cells, named type A pericytes. Type A pericytes (expressing the GLAST-*CreER*^{T2} transgene) undergo tamoxifen-mediated genetic recombination and turn on EYFP expression. Type A pericytes and progeny can be traced by stable and inheritable labeling with

EYFP. (**b**) Distribution of recombined cells (EYFP$^+$) in the adult uninjured spinal cord of GLAST-*CreER^{T2};R26R-EYFP* mice. Type A pericytes are associated with blood vessels throughout the grey and white matter spinal cord parenchyma and meninges surrounding the spinal cord. (**c,d**) Type A pericytes (EYFP$^+$) distribute along capillaries, identified as small caliber blood vessels with transferrin receptor (TFRC)-positive endothelial cells (**c**), and upstream venous and arterial vasculature (**d**), marked by von Willebrand factor (vWF). Insets show magnified boxed regions. Arrowheads point at a recombined cell in a capillary (inset **c**) and in a larger caliber blood vessel (inset **d**). (**e**-**h**) Type A pericytes (EYFP$^+$) encapsulate the endothelial tube (podocalyxin$^+$, Pdx) and express the pan-pericyte markers PDGFRβ (**g**) and CD13 (**h**), but are not labeled by desmin (**e**) and αSMA (**f**), present in other pericytes and vascular smooth muscle cells. (**i**) Detection of *Tbx18* and *Pdgfrb* mRNA in type A pericytes (tdTom$^+$) by RNAscope in situ hybridization combined with immunofluorescence for tdTomato. Arrowheads point at a perivascular tdTom$^+$ cell positive for *Pdgfrb* and *Tbx18* mRNA signals. Endothelial cells are labeled with *Lycopersicon esculentum* (Tomato) lectin. Tbx18, T-box transcription factor 18. Scale bars: 400 μm (**b**), 50 μm (**c**,**d**), 20 μm (**g**), 10 μm (**i**) and 5 μm (**e**,**f**,**h**, close ups in **g**, insets in **c**,**d**). Cell nuclei are labeled with DAPI. All images show coronal sections. Images are representative of three independent experiments.

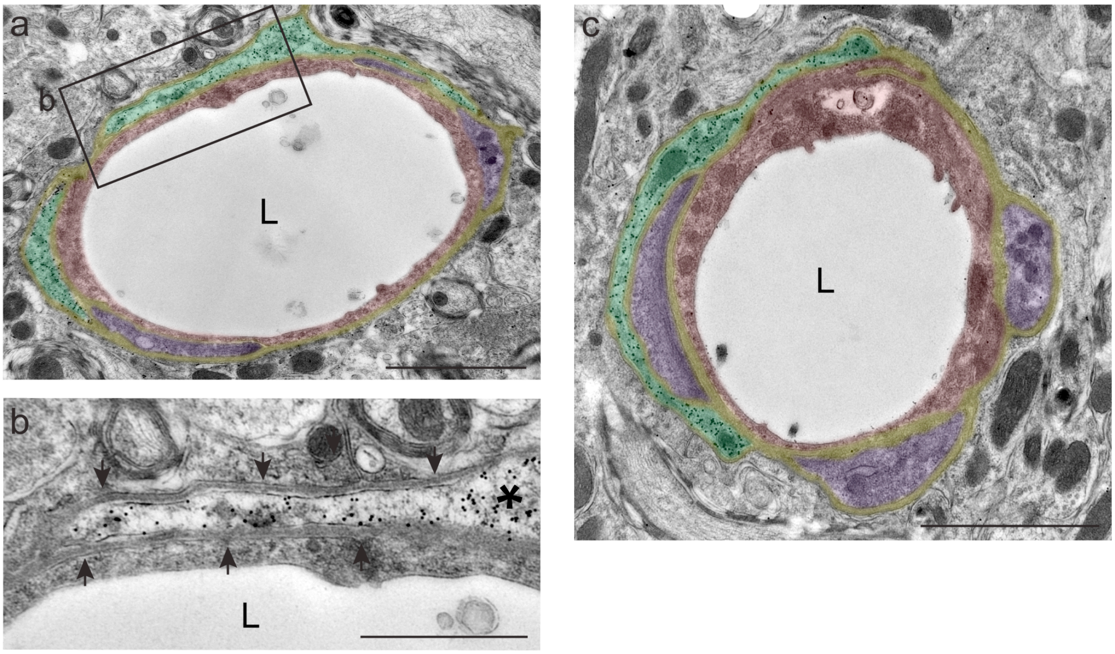

Supplementary Figure 2 | **Electron microscopic visualization of GLAST-expressing type A pericytes in the uninjured mouse spinal cord**

(**a**-**c**) Immunogold electron microscopy showing GLAST-expressing type A pericytes in two different capillaries (**a**,**c**). Ultrathin sections from the uninjured spinal cord of GLAST-*CreER*^{T2}*;R26R-tdTom* mice were incubated with an anti-RFP primary antibody, which recognizes tdTomato, followed by a secondary antibody conjugated with colloidal gold particles. Immunogold particles are specifically localized in GLAST-expressing type A pericytes (tdTom-expressing cells; pseudocolored green). GLAST-expressing pericytes have thin processes, which extend in the pericapillary basal lamina (pseudocolored yellow), where they either face the endothelial cell (pseudocolored red) or other, non-recombined, GLAST-negative pericytes (*i.e.,* type B pericytes; pseudocolored purple). (**b**) depicts a higher magnification of the boxed region in (**a**) showing that the type A pericyte (asterisk) is surrounded by basal lamina (arrows). L indicate the blood vessel lumen. Scale bars: 2 μm (**a**,**c**) and 1 μm (**b**). All images show coronal sections. Images are representative of three independent experiments.

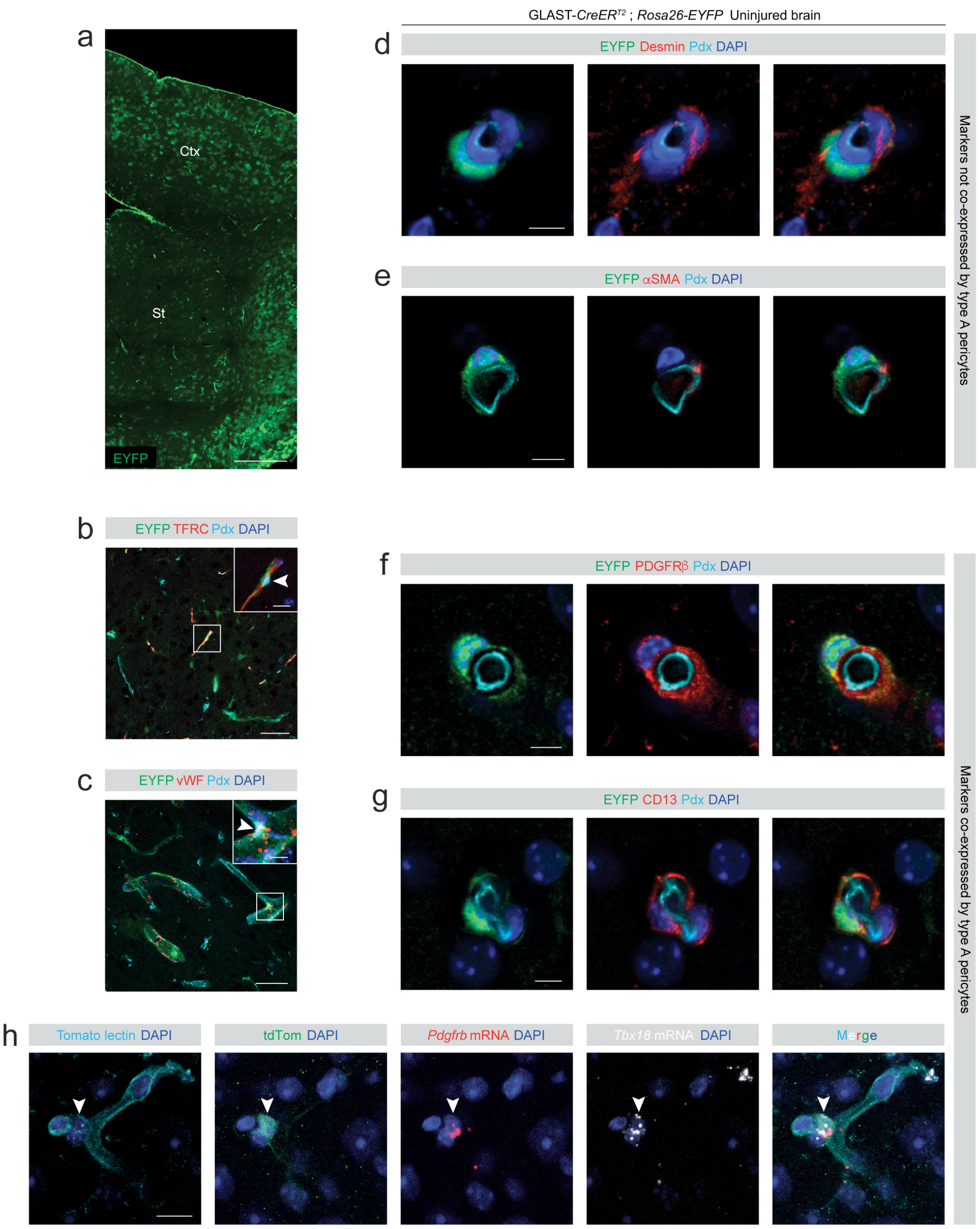

Supplementary Figure 3 | **Genetic labeling of type A pericytes in the adult brain**
(**a**) Distribution of recombined cells (EYFP$^+$) in the adult uninjured forebrain of GLAST-*CreER*^{T2}*;R26R-EYFP* mice. Type A pericytes are associated with blood vessels throughout the cortex (Ctx) and striatum (St) and meninges surrounding the brain. In addition, recombination occurs in parenchymal astrocytes. (**b**,**c**) Type A pericytes (EYFP$^+$) distribute along brain capillaries, identified as small diameter blood vessels with transferrin receptor (TFRC)-positive endothelial cells (**b**), and upstream arteriovenous vasculature (**c**), labeled by von Willebrand factor (vWF). Insets show magnified boxed regions. Arrowheads point at a recombined cell in a capillary (inset **b**) and in a larger caliber blood vessel (inset **c**). Images from the striatum. (**d-g**) Type A pericytes (EYFP$^+$) associate with the endothelial tube (Pdx$^+$)

and express PDGFRβ (**f**) and CD13 (**g**), but are not marked by desmin (**d**) and αSMA (**e**), present in other mural cells in the striatum. (**h**) Detection of *Tbx18* and *Pdgfrb* mRNA in type A pericytes ($tdTom^{+}$) by RNAscope in situ hybridization combined with immunofluorescence for tdTomato. Arrowheads point at a perivascular $tdTom^{+}$ cell positive for *Pdgfrb* and *Tbx18* mRNA signals in the cortex. Endothelial cells are labeled with *Lycopersicon esculentum* (Tomato) lectin. Scale bars: 600 μm (**a**), 50 μm (**b**,**c**), 10 μm (**h**) and 5 μm (**d**-**g**, insets in **b**,**c**). Cell nuclei are labeled with DAPI. All images show coronal sections. Images are representative of three independent experiments.

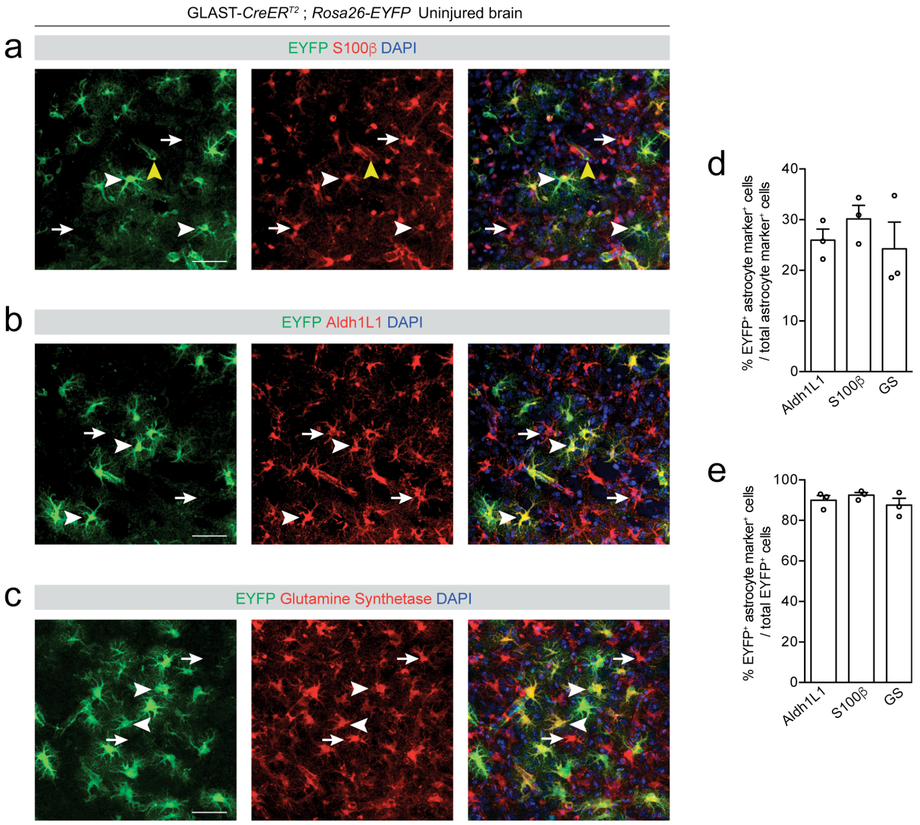

Supplementary Figure 4 | **A subset of astrocytes is recombined in the brain of GLAST-*CreER*^{T2}*;R26R-EYFP* mice**

(**a**-**c**) Recombined parenchymal astrocytes in the uninjured striatum of GLAST-*CreER*^{T2}*;R26R-EYFP* mice express the pan-astrocytic markers S100β (**a**), Aldh1L1 (**b**) and glutamine synthetase (**c**). White arrowheads and arrows point at recombined (EYFP$^+$) and non-recombined (EYFP$^-$) astrocytes, respectively. Yellow arrowheads in (**a**) point at a EYFP$^+$ cell that does not express the astrocyte marker S100β, and represents a type A pericyte. (**d**) Percentage of astrocyte marker$^+$ cells that express EYFP (EYFP$^+$astrocyte marker$^+$ cells) out of total astrocyte marker$^+$ cells. Less than one third of all parenchymal astrocytes are recombined in the striatum of GLAST-*CreER*^{T2}*;R26R-EYFP* mice. (**e**) Percentage of EYFP$^+$ cells that express the astrocyte marker (EYFP$^+$astrocyte marker$^+$ cells) out of total EYFP$^+$ cells. Most recombined cells in the striatal parenchyma of GLAST-*CreER*^{T2}*;R26R-EYFP* mice are astrocytes. The remaining recombined cells, which do not express pan-astrocytic markers, represent type A pericytes. Aldh1L1, aldehyde dehydrogenase 1 family member L1; S100β, S100 calcium binding protein, subunit beta. GS, glutamine synthetase. All scale bars show 50 μm. Data shown as mean ± s.e.m. n=3 animals. Cell nuclei are labeled with DAPI. All images show coronal sections. Images are representative of three independent experiments. Source data and statistical test results are provided as a Source Data file.

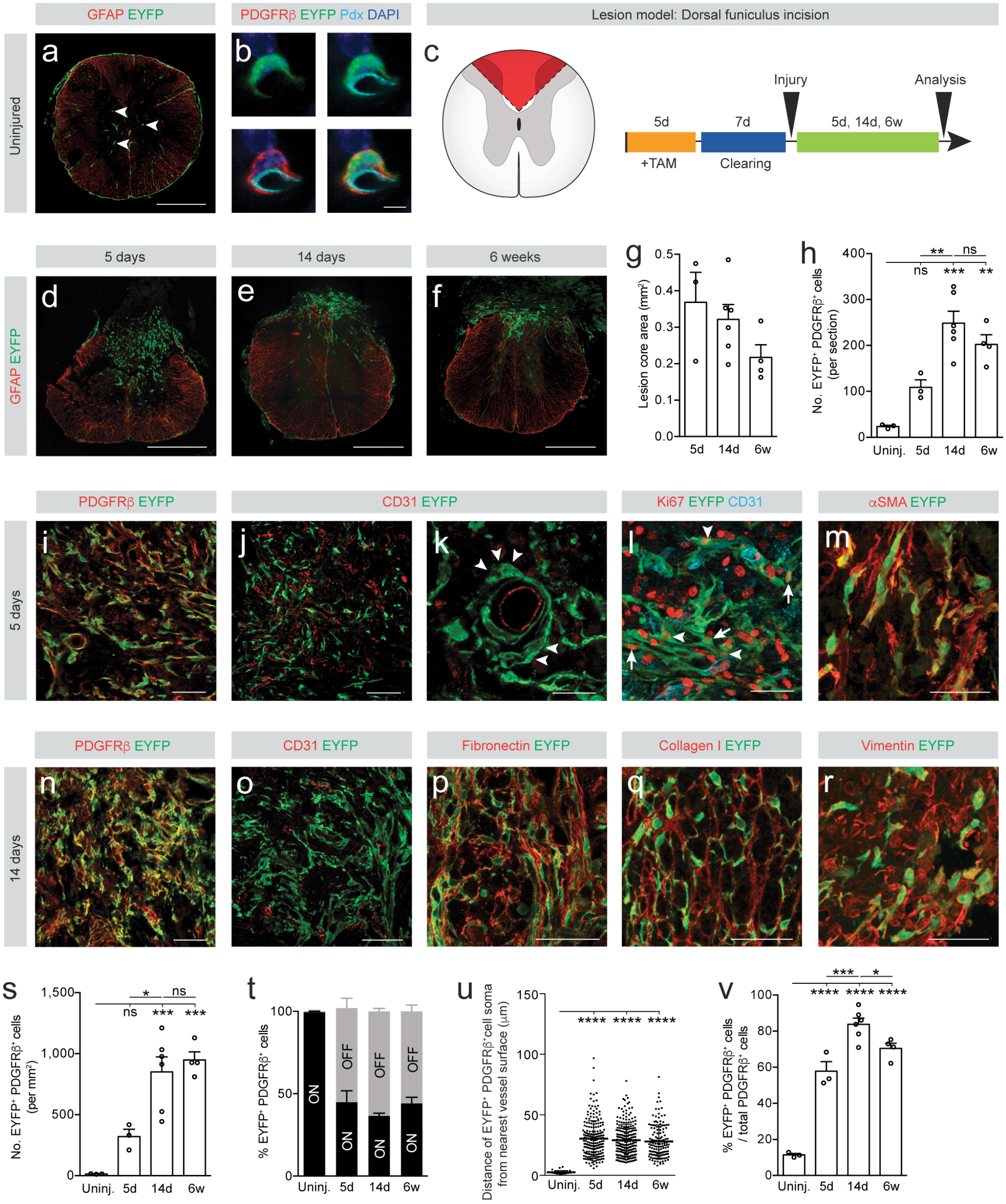

Supplementary Figure 5 | **Type A pericytes are the main source of stromal fibroblasts that form fibrotic scar tissue after penetrating spinal cord injury**
(**a**) A subpopulation of perivascular cells, termed type A pericytes, is recombined (EYFP^{+}, arrowheads) in the uninjured spinal cord of GLAST-*CreER*^{T2}*;R26R-EYFP* mice. Type A pericytes distribute throughout the grey and white matter. (**b**) Type A pericyte (EYFP^{+}) lining the endothelial wall (marked with Podocalyxin, Pdx) and expressing the pericyte marker PDGFRβ (Rβ). (**c**) Injury model and experimental timeline. Red represents the lesion. (**d-f**) Distribution of EYFP^{+} cells at 5 dpi (**d**), 14 dpi (**e**) and 6 wpi (**f**). EYFP^{+} cells cluster in the lesion core and are surrounded by a GFAP^{+} glial scar. (**g**, **h**) Lesion core area (**g**) and number of EYFP^{+}PDGFRβ^{+} cells per section (**h**). (**i-k**) EYFP^{+} cells express the stromal marker PDGFRβ (**i**) and a fraction is located outside the vascular wall (CD31^{+}; arrowheads) at 5 dpi (**j**,**k**). (**l**,**m**)

EYFP⁺ cells proliferate (Ki67⁺) while attached to the blood vessel wall (arrowheads) and away from it (arrows) (**l**), and express the (myo)fibroblast marker αSMA (**m**) at 5 dpi. (**n**,**o**) EYFP⁺ cells retain expression of PDGFRβ (**n**) and a fraction remains outside the vascular wall (**o**) at 14 dpi. (**p**-**r**) EYFP⁺ cells are embedded in fibronectin- (**p**) and collagen I- (**q**) rich ECM (14d pi) and express vimentin at 5 dpi (**r**). (**s**) Density of EYFP⁺PDGFRβ⁺ cells in the fibrotic core. (**t**) Percentage of EYFP⁺ cells that express PDGFRβ (EYFP⁺PDGFRβ⁺ cells) associated with (ON vessel) or located away from (OFF vessel) the vascular wall. (**u**) Distance of EYFP⁺PDGFRβ⁺ cells ON vessel (uninjured spinal cord) or OFF vessel (after injury) from the nearest vessel surface. Each dot represents one cell. (**v**) Percentage of PDGFRβ⁺ cells that express EYFP out of total PDGFRβ⁺ cells. Scale bars: 500 μm (**a**,**d**-**f**), 100 μm (**j**), 50 μm (**i**,**l**-**r**), 20 μm (**k**) and 5 μm (**b**). Data shown as mean ± s.e.m. n=3 (Uninjured), n=3 (5d), n=6 (14d), n=4 (6w) animals in (**g**,**h**,**s**,**t**,**v**); n=34 (Uninjured), n=217 (5d), n=231 (14d) and n=142 (6w) cells examined over 3 animals in (**u**). ns, non-significant; *p<0.05, **p<0.01, ***p<0.001, ****p<0.0001 by One-Way ANOVA followed by Holm-Sidak post-hoc test in (**h**,**s**,**v**) and Kruskal-Wallis test followed by Dunn's post-hoc test in (**u**). Cell nuclei are labeled with DAPI. All images show coronal sections. Images are representative of two independent experiments. Source data and statistical test results are provided as a Source Data file.

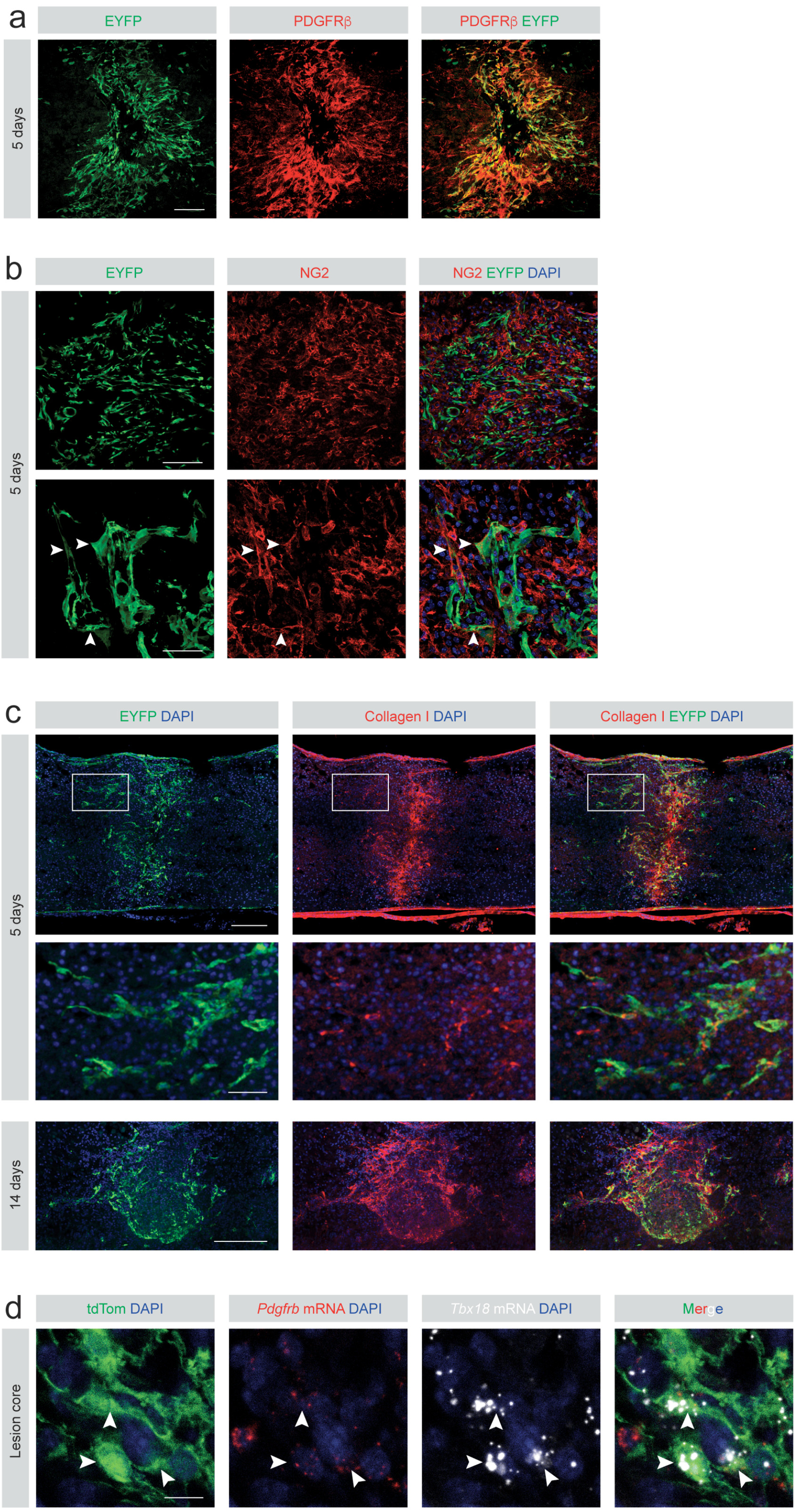

Supplementary Figure 6 | **Characterization of type A pericyte-derived cells after spinal cord injury**

(**a**) Type A pericyte-derived cells (EYFP^{+}) express the stromal marker PDGFRβ at 5 days after complete spinal cord crush. Images show individual channels used to generate the merged image in Fig. 1j. (**b**) While some type A pericytes and progeny

(EYFP$^+$) express the NG2 proteoglycan (lower row, arrowheads), the majority of EYFP$^+$ cells do not express this marker at 5 days after dorsal funiculus incision. (**c**) Type A pericytes and progeny (EYFP$^+$) are embedded in collagen I-positive ECM in the lesion core and surrounding tissue (magnified boxed regions) at 5 and 14 days after complete spinal cord crush. (**d**) Detection of *Tbx18* and *Pdgfrb* mRNA in type A pericyte-derived cells (tdTom$^+$) by RNAscope in situ hybridization combined with immunofluorescence for tdTomato at 14 days after complete spinal cord crush. Arrowheads point at tdTom$^+$ cells positive for *Pdgfrb* and *Tbx18* mRNA signals. Scale bars: 200 µm (**c** upper and lower rows), 100 µm (**a**, **b** upper row), 50 µm (**b** lower row, **c** boxed region) and 10 µm (**d**). Cell nuclei are labeled with DAPI. Images show sagittal (**a**,**c**) and coronal (**b**,**d**) sections. Images are representative of two independent experiments.

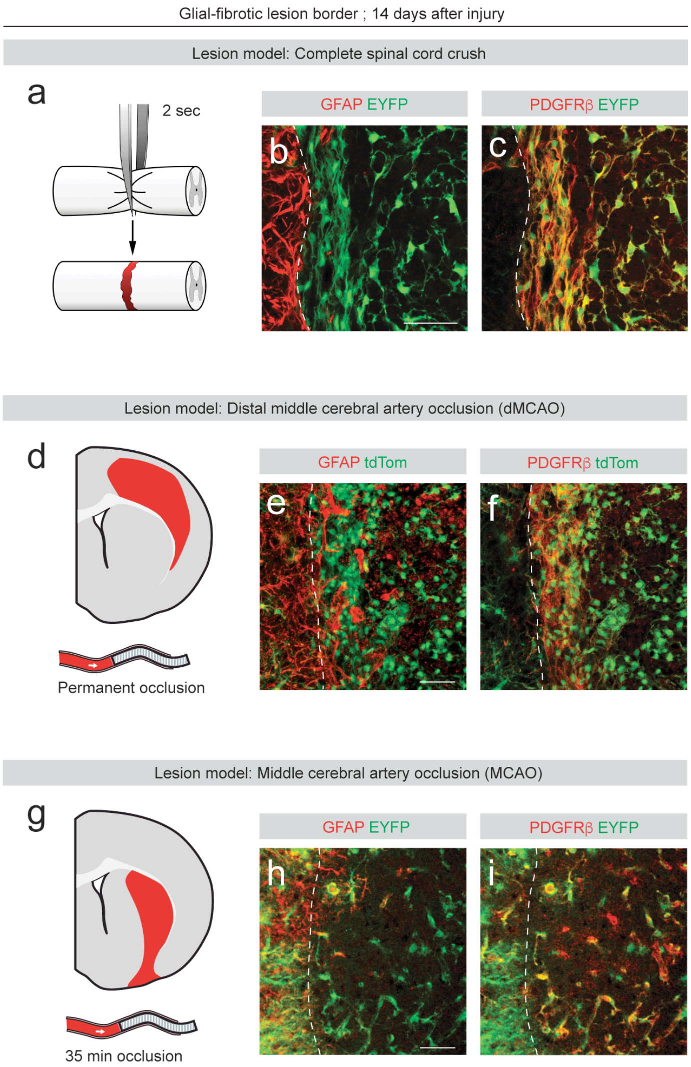

Supplementary Figure 7 | **Type A pericyte-derived cells participate in the formation of the glial-fibrotic lesion border**

(**a-f**) Schematic depictions of the complete spinal cord crush (**a**) and cortical ischemic stroke models (**d**). A sharp and compact border segregating the glial (GFAP$^+$) and fibrotic (type A pericyte-derived, EYFP$^+$ in (**b**) or tdTom$^+$ in (**e**)) compartments of the scar forms at 14 days post-injury (**b**,**e**). Type A pericyte-derived fibroblasts (EYFP$^+$PDGFRβ$^+$ cells in (**c**) or tdTom$^+$PDGFRβ$^+$ cells in (**f**)) aligning with GFAP$^+$ astrocytes along the fibrotic-glial lesion border display distinct morphology than recombined fibroblasts found in the inner core of the fibrotic scar (**c**,**f**). (**g-i**) Schematics depicting the striatal ischemic stroke model (**g**). No formation of an aligned and compact lesion border between GFAP$^+$ astrocytes and type A pericyte-derived cells (EYFP$^+$PDGFRβ$^+$) at 14 days after injury (**h**). EYFP$^+$PDGFRβ$^+$ cells in the inner part of the ischemic stroke core and at the glial-fibrotic lesion border remain attached to the blood vessel wall and display similar morphology (**i**). Red in schematics represents the lesion. All scale bars represent 50 µm. Dashed lines indicate the glial-fibrotic lesion border. (**b**,**c**), (**e**,**f**) and (**h**,**i**) denote paired images. Images show sagittal (**b**,**c**) and coronal (**e**,**f**,**h**,**i**) sections. Images are representative of two independent experiments.

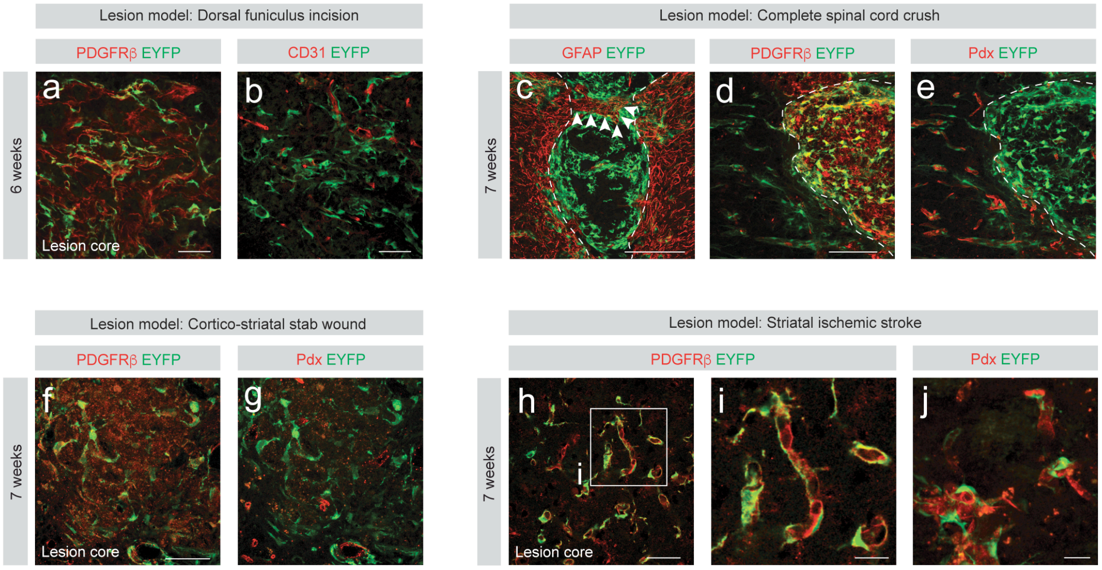

Supplementary Figure 8 | **Type A pericyte-derived cells remain in the lesion core chronically after CNS lesions**

(**a**-**i**) $EYFP^+$ cells, expressing the stromal marker PDGFRβ, persist in the lesion core at 6 weeks after dorsal funiculus incision (**a**), and at 7 weeks after complete spinal cord crush (**d**), cortico-striatal stab wound (**f**) and striatal ischemic stroke (**h**, **i**). For all lesion models (**b**,**e**,**g**), except ischemic stroke lesions confined to the striatum (**j**), most $EYFP^+$ cells are located outside of the vascular wall (labeled with CD31 or podocalyxin, Pdx). The dashed lines in (**c**-**e**) outline the lesion core, flanked by $GFAP^+$ astrocytes in (**c**), and arrowheads point at a glial bridge traversing the lesion core, in a region devoid of recombined cells (**c**). (**i**) shows a higher magnification of the boxed area in (**h**). Scale bars: 200 µm (**c**), 100 µm (**d**,**e**), 50 µm (**a**,**b**,**f**,**g**,**h**) and 20 µm (**i**,**j**). (**d**,**e**) and (**f**,**g**) denote paired images. (**a**,**b**,**h**-**j**) and (**c**-**g**) show coronal and sagittal images, respectively. Images are representative of two independent experiments.

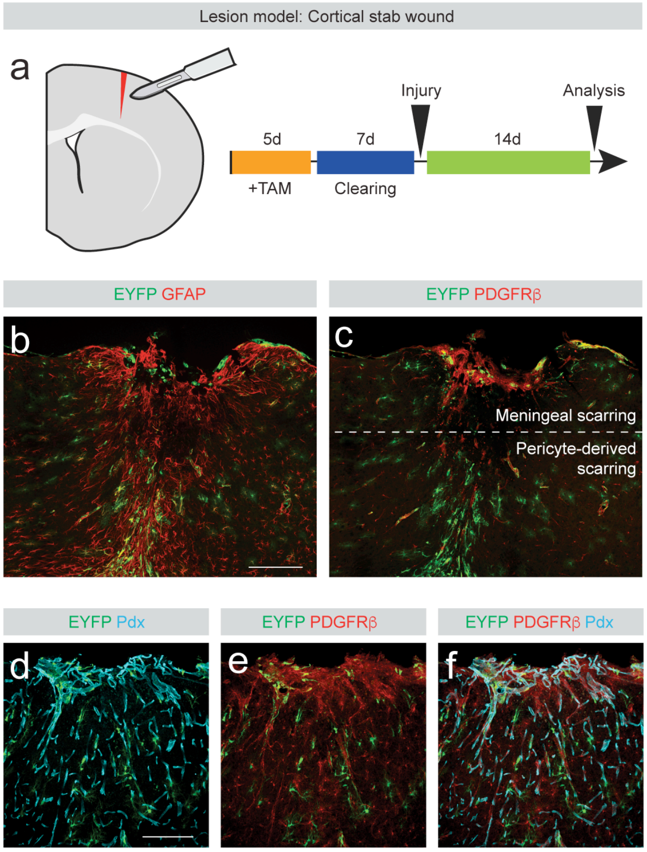

Supplementary Figure 9 | **Small stab lesions restricted to the cerebral cortex do not generate extensive fibrotic scarring**

(**a**) Cortical stab lesion model and experimental timeline. Red represents the lesion. (**b**,**c**) Stab lesions restricted to the cerebral cortex induce widespread gliosis (GFAP^{+}; **b**) and generate meningeal-derived fibrotic scarring close to the brain surface (PDGFRβ^{+}), but do not trigger extensive type A pericyte-derived fibrotic scarring, as observed by limited EYFP^{+}PDGFRβ^{+} cells at 14 dpi (**c**). (**d**-**f**) Recombined cells (EYFP^{+}) do not leave the blood vessel wall (marker by podocalyxin, Pdx) following small cortical stab lesions. Scale bars: 200 μm (**d**-**f**) and 250 μm (**b**,**c**). (**b**,**c**) and (**d**-**f**) show paired images. All images show sagittal sections. Images are representative of two independent experiments.

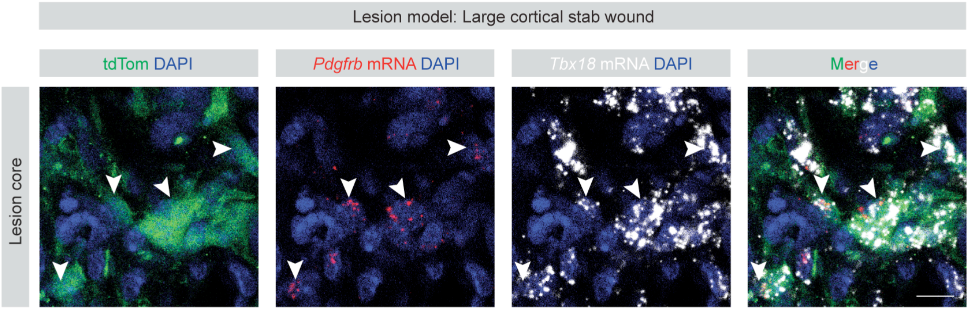

Supplementary Figure 10 | **Type A pericyte-derived cells express Tbx18 after large cortical stab lesions**

Detection of *Tbx18* and *Pdgfrb* mRNA in type A pericyte-derived cells (tdTom^{+}) by RNAscope in situ hybridization combined with immunofluorescence for tdTomato at 5 days after a large cortical stab lesion. Arrowheads point at tdTom^{+} cells positive for *Pdgfrb* and *Tbx18* mRNA signals. Cell nuclei are labeled with DAPI. Scale bar: 10 μm. Images are representative of two independent experiments.

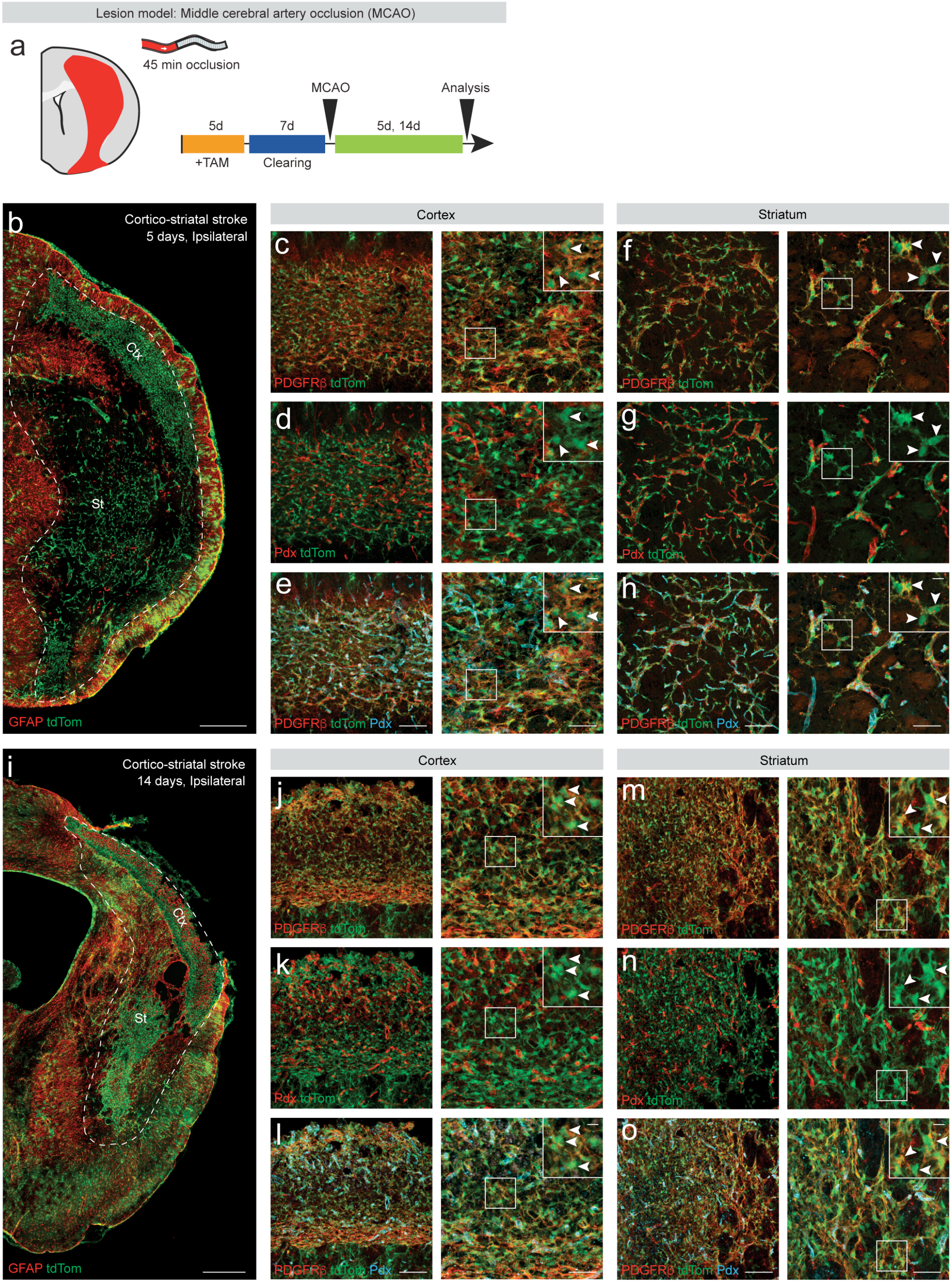

Supplementary Figure 11 | **Type A pericyte-derived cells contribute to stromal fibroblasts after cortico-striatal ischemic stroke**

(**a**) Stroke model and experimental timeline. Red represents the lesion. (**b**) Distribution of recombined (tdTom^{+}) cells at 5 days following ischemia. Partially recombined reactive glial cells (GFAP^{+}) surround the ischemic lesion core (dashed line), filled with tdTom^{+} stromal cells. (**c-h**) TdTom^{+} cells in the ischemic core express the stromal marker PDGFRβ (**c**,**f**) and are located outside of the vascular wall (Pdx^{+}; arrowheads) (**d**,**g**) in the cortex (**c-e**) and striatum (**f-h**) at 5 days post-stroke. (**e**) and (**h**) show merged images of (**c**,**d**) and (**f**,**g**), respectively. (**i**) Distribution of

$tdTom^+$ cells at 14 days after ischemia. Partially recombined reactive glial cells ($GFAP^+$) wall off the ischemic lesion core (dashed line), occupied by $tdTom^+$ type A pericyte-derived stromal cells. (**c-h**) $TdTom^+$ cells in the ischemic stroke core at 14 days after ischemia express PDGFRβ (**j**,**m**) and are found at a distance to blood vessel wall (Pdx^+; arrowheads) (**k**,**n**) in the cortex (**j**-**l**) and striatum (**m**-**o**). (**l**) and (**o**) show merged images of (**j**,**k**) and (**m**,**n**), respectively. St, striatum; Ctx, cortex; Pdx, podocalyxin. Scale bars: 500 μm (**b**,**i**), 100 μm (left panels: **c**-**h**, **j**-**o**), 50 μm (right panels: **c**-**h**, **j**-**o**) and 10 μm (insets). Insets show a higher magnification of boxed areas. All images show coronal sections. Images are representative of two independent experiments.

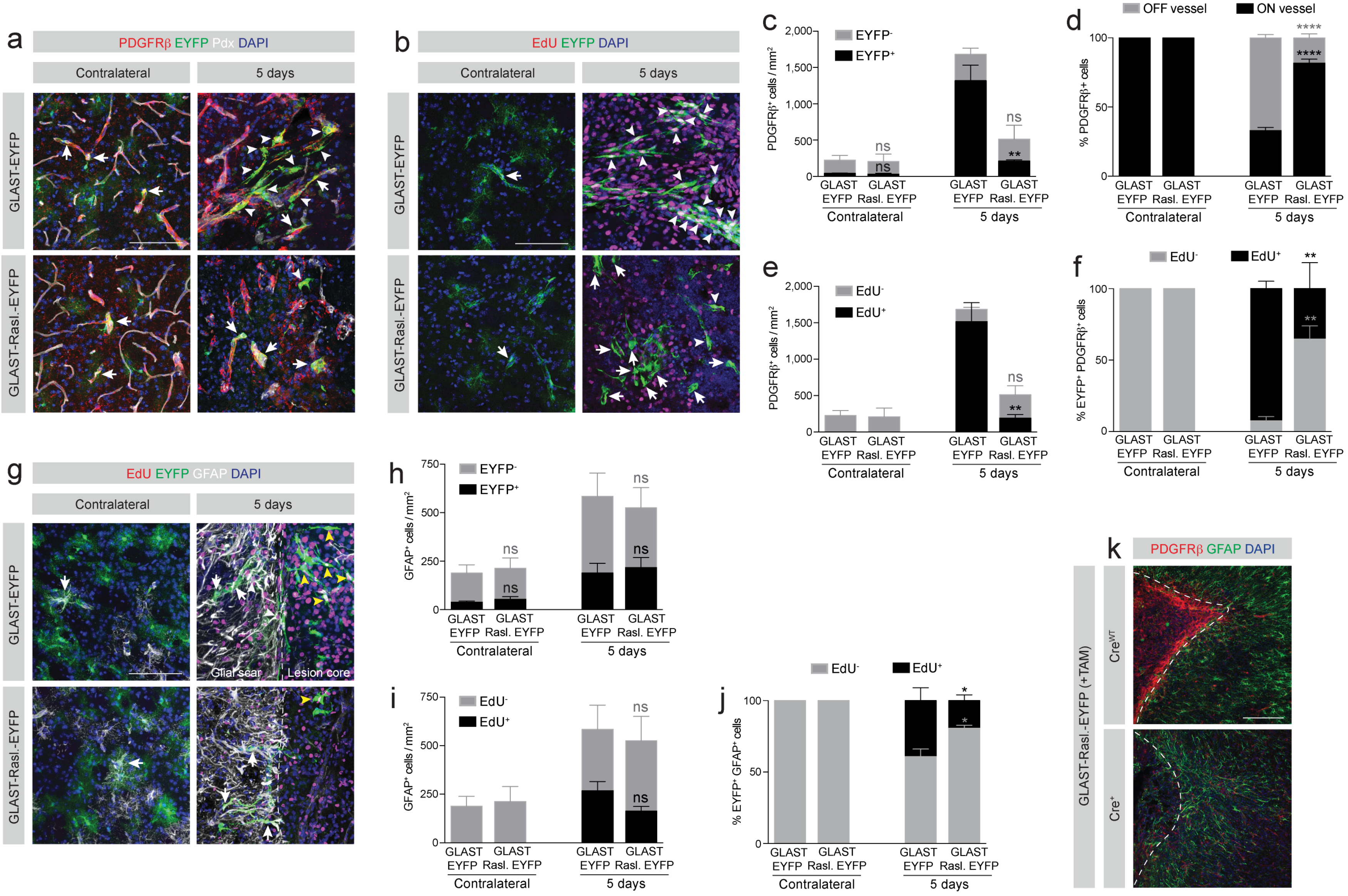
a
PDGFRβ EYFP Pdx DAPI
Contralateral
5 days
GLAST-EYFP
GLAST-Rasl.-EYFP
b
EdU EYFP DAPI
c
EYFP-
EYFP+
PDGFRβ+ cells / mm2
ns
**
GLAST EYFP
GLAST Rasl. EYFP
Contralateral
5 days
d
OFF vessel
ON vessel
% PDGFRβ+ cells
****
e
EdU-
EdU+
f
% EYFP+ PDGFRβ+ cells
g
EdU EYFP GFAP DAPI
Glial scar
Lesion core
h
GFAP+ cells / mm2
i
j
% EYFP+ GFAP+ cells
*
k
PDGFRβ GFAP DAPI
GLAST-Rasl.-EYFP (+TAM)
CreWT
Cre+

Supplementary Figure 12 | **Genetic strategy to modulate the generation of type A pericyte progeny after a cortico-striatal stab wound**

(**a**) Sagittal view of the brain of GLAST-EYFP (also referred to as GLAST-*CreER^{T2};R26R-EYFP*) and GLAST-Rasless-EYFP mice showing type A pericytes (EYFP^{+}PDGFRβ^{+} cells) tightly associated with the vasculature (podocalyxin, Pdx^{+}) in the striatum contralateral to the lesion and type A pericytes and progeny (EYFP^{+}) in the lesion core at 5 days after a cortico-striatal stab wound. Arrowheads and arrows indicate EYFP^{+}PDGFRβ^{+} cells that detached from or remained associated with the vascular wall, respectively. (**b**) Sagittal view of the lesion core and contralateral striatum showing proliferation (EdU incorporation) of recombined pericytes and progeny (EYFP^{+}) in GLAST-EYFP and GLAST-Rasless-EYFP mice. Arrowheads point at EYFP^{+} cells that incorporated EdU. Arrows indicate non-proliferative EYFP^{+} cells. (**c**) Density of recombined (EYFP^{+}) and non-recombined (EYFP^{-}) PDGFRβ^{+} cells. Following injury, the number of recombined type A pericyte-derived stromal cells (EYFP^{+}PDGFRβ^{+}) per area is greatly reduced in GLAST-Rasless-EYFP mice compared to GLAST-EYFP control mice. (**d**) Percentage of PDGFRβ^{+} cells associated with (ON vessel) or dissociated from (OFF vessel) the vascular wall. In the contralateral striatum all PDGFRβ^{+} cells are associated with the vasculature in both GLAST-EYFP and GLAST-Rasless-EYFP mice. After injury, the percentage of PDGFRβ^{+} cells OFF vessel is greatly reduced in GLAST-Rasless-EYFP mice paralleled by a higher percentage of PDGFRβ^{+} cells ON vessel compared to control GLAST-EYFP mice. (**e**) Density of proliferating (EdU^{+}) and non-proliferating (EdU^{-}) PDGFRβ^{+} cells. In the striatum contralateral to the lesion PDGFRβ^{+} cells did not incorporate EdU in neither GLAST-Rasless-EYFP nor GLAST-EYFP mice. Injury-induced proliferation of PDGFRβ^{+} stromal cells is greatly reduced in GLAST-Rasless-EYFP mice compared to control GLAST-EYFP mice. The density of non-proliferating PDGFRβ^{+} stromal cells is not significantly changed. (**f**) Percentage of proliferating (EdU^{+}) and non-proliferating (EdU^{-}) EYFP^{+}PDGFRβ^{+} cells out of all EYFP^{+}PDGFRβ^{+} cells. In the contralateral striatum virtually no EYFP^{+}PDGFRβ^{+} cells incorporate EdU in GLAST-EYFP or GLAST-Rasless-EYFP mice. The proliferation of EYFP^{+}PDGFRβ^{+} cells induced by the injury is greatly reduced in GLAST-Rasless-EYFP mice compared to control GLAST-EYFP mice, resulting in a relative increase in the percentage of non-proliferative EYFP^{+}PDGFRβ^{+} cells. (**g**) Sagittal view of the lesion site and contralateral striatum showing proliferation (EdU incorporation) of EYFP^{+}GFAP^{+} astrocytes in GLAST-YFP and GLAST-Rasless-YFP mice. Proliferating (EdU^{+}) and non-proliferating (EdU^{-}) EYFP^{+}GFAP^{+} astrocytes are indicated by white arrowheads and white arrows, respectively. Yellow arrowheads point at proliferating type A pericyte-derived cells (EYFP^{+}GFAP^{-}) in the lesion core. Dashed lines mark the glial-fibrotic lesion border. (**h**) Density of EYFP^{+} and EYFP^{-} GFAP-expressing astrocytes in the glial scar and contralateral striatum. After injury the number of EYFP^{+}GFAP^{+} astrocytes per area is not significantly changed in GLAST-Rasless-EYFP mice compared to GLAST-EYFP control mice. (**i**) Density of proliferating (EdU^{+}) and non-proliferating (EdU^{-}) GFAP^{+} astrocytes. In the striatum contralateral to the lesion GFAP^{+} cells did not incorporate EdU. Injury-induced proliferation of GFAP^{+} astrocytes is not significantly altered in GLAST-Rasless-EYFP mice compared to control GLAST-EYFP mice. (**j**) Percentage of proliferating (EdU^{+}) and non-proliferating (EdU^{-}) EYFP^{+}GFAP^{+} astrocytes out of all EYFP^{+}GFAP^{+} cells. In the contralateral striatum no EYFP^{+}GFAP^{+} cells incorporate EdU in neither GLAST-EYFP nor GLAST-Rasless-EYFP mice. Injury-induced

proliferation of $EYFP^+GFAP^+$ cells is significantly decreased in GLAST-Rasless-EYFP mice compared to control GLAST-EYFP mice, resulting in a relative increase in the proportion of non-proliferative $EYFP^+GFAP^+$ cells. (**k**) Sagittal view of the lesion site at 14 days after a cortico–striatal stab wound in Cre^{WT} and Cre^+ GLAST-Rasless-EYFP animals. Cre^{WT} control animals develop a larger fibrotic lesion core with increased $PDGFR\beta^+$ scarring when compared to Cre^+ animals. $GFAP^+$ reactive astrocytes border the lesion core. Dashed lines mark the glial-fibrotic lesion border. Scale bars: 200 μm (**k**) and 100 μm (**a**,**b**,**g**). Data shown as mean ± s.e.m. n=3 (GLAST-EYFP contralateral), n=3 (GLAST-EYFP 5 days), n=4 (GLAST-Rasless-EYFP Cre^+ contralateral) and n=4 (GLAST-Rasless-EYFP Cre^+ 5 days) animals. ns, non-significant; *$p<0.05$, **$p<0.01$, ****$p<0.0001$ by two-sided, unpaired Student's t-test. Cell nuclei are labeled with DAPI. Images are representative of two independent experiments. Source data and statistical test results are provided as a Source Data file.

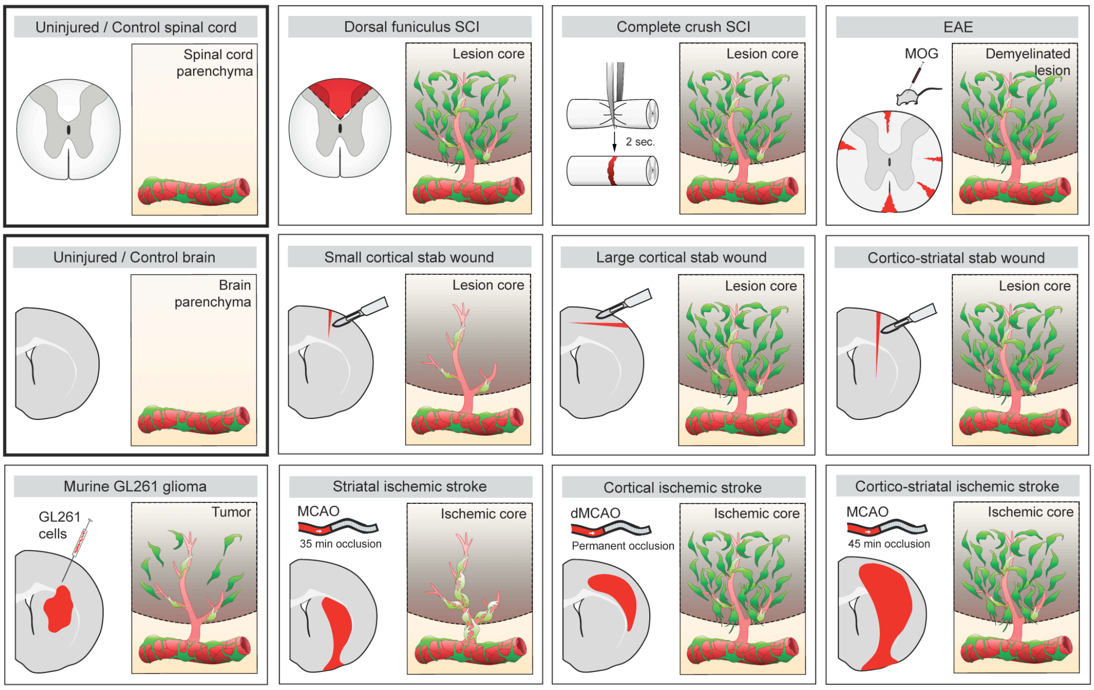

Supplementary Figure 13 | **Pericyte-derived fibrotic scar tissue formation is conserved in response to diverse CNS lesions**

Schematic illustrations depicting the contribution of type A pericyte-derived cells to diverse CNS lesions. In the uninjured/control brain and spinal cord parenchyma, virtually all type A pericytes (cells colored dark green) are found associated with the blood vessel wall. After penetrating and non-penetrating spinal cord injury (SCI), and EAE, type A pericytes are recruited from the vascular wall and give rise to progeny that form fibrotic scar tissue. Small stab lesions restricted to the cerebral cortex do not generate extensive fibrotic scar tissue and, therefore, little to no participation of type A pericytes is observed. However, in larger cortical and cortico-striatal stab lesions, type A pericytes give rise to progeny that locate away from the vascular wall and cluster at the core of the lesion, contributing to fibrotic scar tissue formation. Although the vasculature of murine GL261 gliomas shows reduced coverage by type A pericytes, some recombined PDGFRβ^{+} stromal cells are found in distance to the blood vessel wall. Following ischemic stroke confined to the striatum, type A pericytes and progeny increase in number but remain associated with the vascular wall. In contrast, cortical and cortico-striatal ischemic lesions trigger type A pericyte recruitment from the blood vessel wall and generation of fibrotic scar tissue by pericyte-derived cells. Light colored regions in the scheme represent peri-lesion tissue and darker regions indicate the lesion. Cells colored light green in the lesion represent type A pericytes and progeny that remain associated with the vascular wall, whereas cells colored dark green in the lesion represent type A pericytes and progeny that no longer associate with the blood vessel. MCAO, middle cerebral artery occlusion (MCA); dMCAO, occlusion of the distal portion of the MCA.

Supplementary Table 1 | Clinical and neuropathological data of Spinal Cord Injury patients

Case ID	Injury level*	AIS grade	Injury-death interval (days)	Tissue harvest post mortem (hrs)	Type of Injury#	Diagnosis
BB1	C6	A	10	26	MC	Three column fracture dislocation
BB3	C6	B	17	49	MC	Bilateral facet dislocation
BB4	C7	A	34	14	MC	Three column fracture dislocation
BB5	C6	A	9	13	MC	Bilateral facet dislocation
BB9	C4	C	16	26	C/C	Hyperextension, avulsion flakes, traumatic disc
BB6	C4	D	61	15	C/C	Hyperextension, avulsion flakes, traumatic disc

* Cervical ; Number indicates vertebrae
# MC – Massive compression type injury ; C/C – Contusion/Cyst type injury

Supplementary Table 2 | Clinical and neuropathological data of Multiple Sclerosis patients

Case ID	Disease type*	Disease duration (years)	Lesion stage‡	Tissue harvest post mortem (hrs)	Diagnosis
MS 058	SPMS	21	AL / CAL	15	Chronic MS
MS 062	SPMS	19	AL / CAL	10	Chronic MS
MS 066	ND	56	CAL	21	Chronic MS
MS 074	SPMS	36	CAL	7	Chronic MS ; Significant demyelination
MS 092	SPMS	17	CAL	26	Chronic MS ; Epileptic seizures
MS 097	SPMS	22	CAL	31	Chronic MS
C14	NA	NA	NA	18	Control tissue (spinal cord looks normal)
C37	NA	NA	NA	5	Control tissue (spinal cord looks normal)
C39	NA	NA	NA	21	Control tissue (spinal cord looks normal)
C43	NA	NA	NA	12	Control tissue (spinal cord looks normal)

* SPMS – Secondary Progressive Multiple Sclerosis ; ND – Not documented (little medical history available)
‡ AL – Active Lesion ; CAL – Chronic Active Lesion
NA – Non applicable

Supplementary Table 3 | Clinical and neuropathological data of Stroke patients

Case ID	Type of stroke	Location of stroke	Size of infarction (cm^3)	Stroke-death interval	Tissue harvest post mortem (hrs)	Cause of infarction
16-573	Territorial (Arteria cerebri media)	Right frontal cortex	≈ 36	3 months	36	Atrial fibrillation (cardiac source)
14-24	Territorial (Arteria cerebri media)	Right Basal ganglia (including thalamus)	≈ 26	7 weeks	24	Stenosis of Internal carotid artery (NASCET 80%*)
14-16	Lacunar	Left striatum	≈ 2	4 weeks	36	Artherothrombotic
13-69	(i) Lacunar ; (ii) Territorial (Arteria cerebri media)	(i) Left striatum (ii) Left parietal cortex	(i) ≈ 2 (ii) ≈ 48	(i) 31 days (ii) 26 months	36	Atrial fibrillation (cardiac source)

*NASCET – North American Symptomatic Carotid Endarterectomy Trial

Supplementary Table 4 | Clinical and neuropathological data of Glioblastoma patients

Case ID	Type of tumor	Location of tumor	Tumor size (cc)
FP190138	Glioblastoma (WHO Grade IV; Astrocytoma)	Left temporal lobe	≈ 54
FP220453	Glioblastoma (WHO Grade IV; Astrocytoma)	Left fronto-temporal lobe	≈ 60
FP070741	Glioblastoma (WHO Grade IV; Astrocytoma)	Corpus callosum spreading bihemispherically with dominance on the right side	≈ 62
