# Supplementary material for: Pericyte-derived fibrotic scarring is conserved across diverse central nervous system lesions
Source: Nat Commun. 2021 Sep 17;12:5501. doi: 10.1038/s41467-021-25585-5 (PMC8448846; doi:10.1038/s41467-021-25585-5)
Supplement: Supplementary file 3 — Reporting Summary [file 41467_2021_25585_MOESM3_ESM.pdf]

nature research

Corresponding author(s): Christian Goeritz

Last updated by author(s): Jul 8, 2021

Reporting Summary

Nature Research wishes to improve the reproducibility of the work that we publish. This form provides structure for consistency and transparency in reporting. For further information on Nature Research policies, see Authors & Referees and the Editorial Policy Checklist.

Statistics

For all statistical analyses, confirm that the following items are present in the figure legend, table legend, main text, or Methods section.

n/a	Confirmed	
☐	☒	The exact sample size (*n*) for each experimental group/condition, given as a discrete number and unit of measurement
☐	☒	A statement on whether measurements were taken from distinct samples or whether the same sample was measured repeatedly
☐	☒	The statistical test(s) used AND whether they are one- or two-sided<br>*Only common tests should be described solely by name; describe more complex techniques in the Methods section.*
☐	☒	A description of all covariates tested
☐	☒	A description of any assumptions or corrections, such as tests of normality and adjustment for multiple comparisons
☐	☒	A full description of the statistical parameters including central tendency (e.g. means) or other basic estimates (e.g. regression coefficient) AND variation (e.g. standard deviation) or associated estimates of uncertainty (e.g. confidence intervals)
☐	☒	For null hypothesis testing, the test statistic (e.g. *F*, *t*, *r*) with confidence intervals, effect sizes, degrees of freedom and *P* value noted<br>*Give P values as exact values whenever suitable.*
☒	☐	For Bayesian analysis, information on the choice of priors and Markov chain Monte Carlo settings
☒	☐	For hierarchical and complex designs, identification of the appropriate level for tests and full reporting of outcomes
☒	☐	Estimates of effect sizes (e.g. Cohen's *d*, Pearson's *r*), indicating how they were calculated

Our web collection on statistics for biologists contains articles on many of the points above.

Software and code

Policy information about availability of computer code

Data collection	Immunofluorescence images were acquired with a Leica DM5500 B bright-field microscope coupled to the LAS X 3.7.2.22383 software, a Zeiss Axioplan 2 upright epifluorescent microscope equipped with the ZEN 2 software (version 2.0.14283.302) or a Leica TCS SP8X confocal microscope equipped with the LAS X 3.5.7.23225 software. Electron microscopic images were acquired with a Tecnai 12 electron microscope (FEI) at 80 kV equipped with ITEM FEI version 5.1 software (Olympus Soft Imaging Solutions, Münster, Germany).
Data analysis	Image analysis, processing, assembly and cell counting were performed with the open source software ImageJ/Fiji (version 2.0.0-rc-43/1.51j for Mac), Adobe Photoshop CC 2018 19.1.1 release and Illustrator CC 2018 22.0.1 release for Mac. GraphPad Prism version 6.0g software for Mac was used for all statistical analysis.

For manuscripts utilizing custom algorithms or software that are central to the research but not yet described in published literature, software must be made available to editors/reviewers. We strongly encourage code deposition in a community repository (e.g. GitHub). See the Nature Research guidelines for submitting code & software for further information.

Data

Policy information about availability of data

All manuscripts must include a data availability statement. This statement should provide the following information, where applicable:

- Accession codes, unique identifiers, or web links for publicly available datasets
- A list of figures that have associated raw data
- A description of any restrictions on data availability

The authors declare that all data supporting the findings of this study are included in this published article (and its supplementary information files). Source Data for Figures 1–8 and Supplementary Figures 4, 5 and 12 are provided with the paper.

Field-specific reporting

Please select the one below that is the best fit for your research. If you are not sure, read the appropriate sections before making your selection.

☒ Life sciences ☐ Behavioural & social sciences ☐ Ecological, evolutionary & environmental sciences

For a reference copy of the document with all sections, see nature.com/documents/nr-reporting-summary-flat.pdf

Life sciences study design

All studies must disclose on these points even when the disclosure is negative.

Sample size	No statistical methods have been used to predetermine sample size. Based on our previous work (Dias, D. O. et al. Reducing Pericyte-Derived Scarring Promotes Recovery after Spinal Cord Injury. Cell 173, 153-165.e22 (2018); Göritz, C. et al. A pericyte origin of spinal cord scar tissue. Science (80-.). 333, (2011)), groups of 3 to 4 animals are sufficient for quantitative analyses, but in most cases, more animals were used.
Data exclusions	As pre-established, for complete crush spinal cord injury experiments, animals undergoing surgery that lost more than 15% of their pre-operative body-weight were euthanized.
Replication	Replicate measurements were obtained by analyzing 3 or more alternate tissue sections per animal or human biospecimen. Three or more biological replicates were used per condition. All attempts at replication were successful.
Randomization	All animals used in experiments involving crush spinal cord injury were female mice with approximately similar age, weight and genetic background. All animals used in experiments involving ischemic stroke (MCAO model) were male mice with approximately similar age, weight and genetic background. For all other experiments, both male and female mice of similar age, weight and genetic background were equally distributed across experimental groups.<br>For analyses involving human biospecimens, samples from control donor individuals or donor individuals with the disease of interest were obtained from biobanks or collaborators. Due to limited sample availability, all samples were analysed.
Blinding	The investigators were blinded to group allocation during data collection and analyses.

Reporting for specific materials, systems and methods

We require information from authors about some types of materials, experimental systems and methods used in many studies. Here, indicate whether each material, system or method listed is relevant to your study. If you are not sure if a list item applies to your research, read the appropriate section before selecting a response.

Materials & experimental systems

n/a	Involved in the study	
☐	☒	Antibodies
☐	☒	Eukaryotic cell lines
☒	☐	Palaeontology
☐	☒	Animals and other organisms
☐	☒	Human research participants
☒	☐	Clinical data

Methods

n/a	Involved in the study	
☒	☐	ChIP-seq
☒	☐	Flow cytometry
☒	☐	MRI-based neuroimaging

Antibodies

Antibodies used	The following primary antibodies were used for immunohistochemistry: GFAP (1:1000, chicken, Millipore, AB5541), GFAP (1:200, guinea pig, Synaptic Systems, 173004), GFAP (1:1000, mouse directly conjugated to Cy3, Sigma-Aldrich, C9205, clone G-A-5), GFAP (1:200, rabbit, Dako, Z0334), Aldh1L1 (1:500, rabbit, Abcam, ab87117), S100β (1:500, guinea pig, Synaptic Systems, 287004), glutamine synthetase (1:2000, rabbit, Invitrogen, PA1-46165), PDGFRβ (1:200, rabbit, Abcam, ab32570, clone Y92), PDGFRβ (1:100, rat, eBioscience, 14-1402-82, clone APB5), PDGFRβ (1:200, goat, R&D Systems, AF1042), PDGFRβ (1:100, goat, R&D Systems, AF385), CD31 (1:100, mouse, Dako, M0823, clone JC70A), CD31 (1:100, rat, BD Biosciences, 550274, clone MEC 13.3), podocalyxin (1:200, goat, R&D Systems, AF1556), von Willebrand factor (1:200, rabbit, Dako, A0082), MBP (1:1000, rat, AbD Serotec, MCA409S, clone 12), MBP (1:1000, mouse, Covance, SMI-94R, clone SMI-94), NG2 (1:200, rabbit, Millipore, AB5320), GFP (1:2000, goat directly conjugated to FITC, Abcam, ab6662), GFP (1:10000, chicken, Aves Labs, GFP-1020), GFP (1:500, sheep, Bio-Rad, 4745-1051), Red Fluorescent Protein (1:500, rabbit, Rockland, 600-401-379), Red Fluorescent Protein (1:500, chicken, Novus Biologicals, NBP1-97371), fibronectin (1:500, sheep, Bio-Rad, 44470-2304), collagen type I (1:100, goat, Southern Biotech, 1310-01), collagen type I (1:200, rabbit, Abcam, ab21286), CD13 (1:500, rat, Abcam, ab33489, clone R3-63), CD45 (1:500, rabbit, Abcam, ab10558), CD3 (1:200, rat, Biolegend, 100201, clone 17A2), TFRC (1:200, mouse, Thermo Fisher Scientific, 13-6800, clone H68.4), TFRC (1:200, rat, Novus Biologicals, NB100-64979, clone 8D3), Ki67 (1:1000, rat, eBioscience, 14-5698, clone SolA15), vimentin (1:1000, chicken, Chemicon, AB5733), vimentin (1:500, rabbit, Abcam, ab92547, clone EPR3776), desmin (1:200, rabbit, Abcam, ab15200), αSMA (1:500, mouse directly conjugated to Cy3, Sigma-Aldrich, C6198, clone 1A4) and αSMA (1:100, rabbit, Abcam, ab5694). UEA-1 (1:200, directly conjugated to FITC or Rhodamine, Vector Labs, FL-1061 or

RL-1062, respectively) was used to label human endothelial cells. Biotinylated Lycopersicon Esculentum (Tomato) lectin (biotinylated, Vector Labs, B-1175-1) was used to label blood vessels in mouse tissue. FluoroMyelin Red fluorescent myelin stain (1:500, Invitrogen, F34652) was used to label myelin in mouse tissue.

The following secondary antibody was used for immunogold cytochemistry (electron microscopy): goat anti-rabbit IgG conjugated to 15 nm gold particles (1:20, Abcam, ab27236).
All secondary antibodies used for immunohistochemistry were F(ab')2 fragment affinity-purified antibodies purchased from Jackson Immunoresearch and diluted at 1:500: DyLight 405 donkey anti-chicken IgY (703-476-155), Alexa Fluor 488 donkey anti-chicken IgY (703-546-155), Cy3 donkey anti-chicken IgY (703-166-155), Alexa Fluor 647 donkey anti-chicken IgY (703-606-155), Alexa Fluor 488 donkey anti-goat IgG (705-546-147), Alexa Fluor 488 donkey anti-goat IgG (705-166-147), Alexa Fluor 647 donkey anti-goat IgG (705-606-147), Cy3 donkey anti-guinea pig IgG (706-166-148), Alexa Fluor 647 donkey anti-guinea pig IgG (706-606-148), Cy3 donkey anti-mouse IgG (715-166-151), Alexa Fluor 647 donkey anti-mouse IgG (715-606-151), Alexa Fluor 488 donkey anti-rabbit IgG (711-546-152), Cy3 donkey anti-rabbit IgG (711-166-152), Alexa Fluor 647 donkey anti-rabbit IgG (711-606-152), Alexa Fluor 488 donkey anti-rat IgG (712-546-153), Cy3 donkey anti-rat IgG (712-166-153), Alexa Fluor 647 donkey anti-rat IgG (712-606-153), Alexa Fluor 488 donkey anti-sheep IgG (713-546-147), Cy3 donkey anti-sheep IgG (713-166-147), biotin-SP (long spacer) donkey anti-goat IgG (705-066-147) and biotin-SP (long spacer) donkey anti-rat IgG (712-066-153). Biotinylated secondary antibodies were revealed with Alexa Fluor 488 conjugated Streptavidin (1:500, Jackson Immunoresearch, 016-540-084) or Cy3 conjugated Streptavidin (1:500, Jackson Immunoresearch, 016-160-084).

Validation

Only previously published antibodies or antibodies with company based validations were used for immunohistochemistry:

GFAP (1:1000, chicken, Millipore, AB5541): Dias, D. O. et al. Reducing Pericyte-Derived Scarring Promotes Recovery after Spinal Cord Injury. Cell 173, 153-165.e22 (2018); https://www.merckmillipore.com/SE/en/product/Anti-Glial-Fibrillary-Acidic-Protein-Antibody,MM_NF-AB5541

GFAP (1:200, guinea pig, Synaptic Systems, 173004): Dias, D. O. et al. Reducing Pericyte-Derived Scarring Promotes Recovery after Spinal Cord Injury. Cell 173, 153-165.e22 (2018); https://sysy.com/product/173004

GFAP (1:1000, mouse directly conjugated to Cy3, Sigma-Aldrich, C9205, clone G-A-5): Dias, D. O. et al. Reducing Pericyte-Derived Scarring Promotes Recovery after Spinal Cord Injury. Cell 173, 153-165.e22 (2018); https://www.sigmaaldrich.com/SE/en/product/sigma/c9205

GFAP (1:200, rabbit, Dako, Z0334): Buss, A. et al. TGF-β1 and TGF-β2 expression after traumatic human spinal cord injury. Spinal Cord 46, 364–371 (2008); https://www.agilent.com/en/product/immunohistochemistry/antibodies-controls/primary-antibodies/glial-fibrillary-acidic-protein-(concentrate)-76683#references

Aldh1L1 (1:500, rabbit, Abcam, ab87117): Clavreul, S. et al. Cortical astrocytes develop in a plastic manner at both clonal and cellular levels. Nat. Commun. 10, 4884 (2019)

S100β (1:500, guinea pig, Synaptic Systems, 287004): Roberts, B. M. et al. GABA uptake transporters support dopamine release in dorsal striatum with maladaptive downregulation in a parkinsonism model. Nat. Commun. 11, 4958 (2020); https://sysy.com/product/287004#list

Glutamine synthetase (1:2000, rabbit, Invitrogen, PA1-46165): https://www.thermofisher.com/antibody/product/Glutamine-Synthetase-Antibody-Polyclonal/PA1-46165

PDGFRβ (1:200, rabbit, Abcam, ab32570, clone Y92): Göritz, C. et al. A pericyte origin of spinal cord scar tissue. Science (80-.). 333, (2011); Dias, D. O. et al. Reducing Pericyte-Derived Scarring Promotes Recovery after Spinal Cord Injury. Cell 173, 153-165.e22 (2018)

PDGFRβ (1:100, rat, eBioscience, 14-1402-82, clone APB5): Vanlandewijck, M. et al. A molecular atlas of cell types and zonation in the brain vasculature. Nature 554, 475–480 (2018); Diéguez-Hurtado, R. et al. Loss of the transcription factor RBPJ induces disease-promoting properties in brain pericytes. Nat. Commun. 10, 2817 (2019).

PDGFRβ (1:200, goat, R&D Systems, AF1042): Diéguez-Hurtado, R. et al. Loss of the transcription factor RBPJ induces disease-promoting properties in brain pericytes. Nat. Commun. 10, 2817 (2019).

PDGFRβ (1:100, goat, R&D Systems, AF385): Craggs, L. J. L., Fenwick, R., Oakley, A. E., Ihara, M. & Kalaria, R. N. Immunolocalization of platelet-derived growth factor receptor-β (PDGFR-β) and pericytes in cerebral autosomal dominant arteriopathy with subcortical infarcts and leukoencephalopathy (CADASIL). Neuropathol. Appl. Neurobiol. 41, 557–570 (2015); https://www.rndsystems.com/products/human-pdgf-rbeta-antibody_af385?utm_source=labome&utm_medium=referral&utm_campaign=product_AF385&utm_term=primaryantibodies&utm_content=editorial

CD31 (1:100, mouse, Dako, M0823, clone JC70A): Buss, A. et al. TGF-β1 and TGF-β2 expression after traumatic human spinal cord injury. Spinal Cord 46, 364–371 (2008); https://www.agilent.com/en/product/immunohistochemistry/antibodies-controls/primary-antibodies/cd31-endothelial-cell-(dako-omnis)-76224

CD31 (1:100, rat, BD Biosciences, 550274, clone MEC 13.3): Dias, D. O. et al. Reducing Pericyte-Derived Scarring Promotes Recovery after Spinal Cord Injury. Cell 173, 153-165.e22 (2018); Henderson, N. C. et al. Targeting of αv integrin identifies a core molecular pathway that regulates fibrosis in several organs. Nat. Med. 19, 1617–1624 (2013).

CD13 (1:500, rat, Abcam, ab33489, clone R3-63): Göritz, C. et al. A pericyte origin of spinal cord scar tissue. Science (80-.). 333, (2011); https://www.abcam.com/cd13-antibody-r3-63-ab33489.html?productWallTab=Abreviews

Podocalyxin (1:200, goat, R&D Systems, AF1556): Dias, D. O. et al. Reducing Pericyte-Derived Scarring Promotes Recovery after Spinal Cord Injury. Cell 173, 153-165.e22 (2018); Vanlandewijck, M. et al. A molecular atlas of cell types and zonation in the brain vasculature. Nature 554, 475–480 (2018); Diéguez-Hurtado, R. et al. Loss of the transcription factor RBPJ induces disease-promoting properties in brain pericytes. Nat. Commun. 10, 2817 (2019); https://www.rndsystems.com/products/mouse-podocalyxin-antibody_af1556#product-details

von Willebrand factor (1:200, rabbit, Dako, A0082): Göritz, C. et al. A pericyte origin of spinal cord scar tissue. Science (80-.). 333, (2011); Mezey, É. et al. An immunohistochemical study of lymphatic elements in the human brain. Proc. Natl. Acad. Sci. 118, e2002574118 (2021).; https://www.agilent.com/en/product/immunohistochemistry/antibodies-controls/primary-antibodies/von-willebrand-factor-(concentrate)-76122#specifications

MBP (1:1000, rat, AbD Serotec, MCA409S, clone 12): Yeung, M. S. Y. et al. Dynamics of oligodendrocyte generation and myelination in the human brain. Cell 159, 766–774 (2014); https://www.bio-rad-antibodies.com/monoclonal/cow-bovine-mbp-antibody-12-mca409.html?f=s%2Fn

MBP (1:1000, mouse, Covance, SMI-94R, clone SMI-94): https://www.biolegend.com/de-at/products/purified-anti-myelin-basic-protein-antibody-13166

NG2 (1:200, rabbit, Millipore, AB5320): Diéguez-Hurtado, R. et al. Loss of the transcription factor RBPJ induces disease-promoting properties in brain pericytes. Nat. Commun. 10, 2817 (2019); https://www.merckmillipore.com/SE/en/product/Anti-NG2-Chondroitin-Sulfate-Proteoglycan-Antibody,MM_NF-AB5320#overview

GFP (1:10000, chicken, Aves Labs, GFP-1020): Dias, D. O. et al. Reducing Pericyte-Derived Scarring Promotes Recovery after Spinal Cord Injury. Cell 173, 153-165.e22 (2018); https://www.aveslabs.com/products/anti-green-fluorescent-protein-antibody-gfp

GFP (1:500, sheep, Bio-Rad, 4745-1051): Haberlandt, C. et al. Gray Matter NG2 Cells Display Multiple Ca2+-Signaling Pathways and Highly Motile Processes. PLoS One 6, e17575 (2011); https://www.bio-rad-antibodies.com/polyclonal/green-fluorescent-protein-antibody-4745-1051.html?f=purified

GFP (1:2000, goat directly conjugated to FITC, Abcam, ab6662): Göritz, C. et al. A pericyte origin of spinal cord scar tissue. Science (80-.). 333, (2011); https://www.abcam.com/fitc-gfp-antibody-ab6662.html

Red Fluorescent Protein (1:500, rabbit, Rockland, 600-401-379): Anderson, M. A. et al. Required growth facilitators propel axon regeneration across complete spinal cord injury. Nature 561, 396–400 (2018); https://rockland-inc.com/store/Antibodies-to-GFP-and-Antibodies-to-RFP-600-401-379-O4L_24299.aspx

Red Fluorescent Protein (1:500, chicken, Novus Biologicals, NBP1-97371): Anderson, M. A. et al. Required growth facilitators propel axon regeneration across complete spinal cord injury. Nature 561, 396–400 (2018); https://www.novusbio.com/products/rfp-antibody_nbp1-97371

Fibronectin (1:500, sheep, Bio-Rad, 44470-2304): https://www.bio-rad-antibodies.com/polyclonal/human-fibronectin-antibody-vpa00045.html

Collagen type I (1:100, goat, Southern Biotech, 1310-01): Ke, Q. et al. UCP2-induced hypoxia promotes lipid accumulation and tubulointerstitial fibrosis during ischemic kidney injury. Cell Death Dis. 11, 26 (2020)

Collagen type I (1:200, rabbit, Abcam, ab21286): Dias, D. O. et al. Reducing Pericyte-Derived Scarring Promotes Recovery after Spinal Cord Injury. Cell 173, 153-165.e22 (2018); https://www.abcam.com/collagen-i-antibody-ab21286.html

CD45 (1:500, rabbit, Abcam, ab10558): Göritz, C. et al. A pericyte origin of spinal cord scar tissue. Science (80-.). 333, (2011); Gerzanich, V. et al. Salutary effects of glibenclamide during the chronic phase of murine experimental autoimmune encephalomyelitis. J. Neuroinflammation 14, 177 (2017); Makar, T. K. et al. Silencing of Abcc8 or inhibition of newly upregulated Sur1-Trpm4 reduce inflammation and disease progression in experimental autoimmune encephalomyelitis. J. Neuroinflammation 12, 210 (2015).

CD3 (1:200, rat, Biolegend, 100201, clone 17A2): https://www.biolegend.com/en-us/products/purified-anti-mouse-cd3-antibody-48; Sargent, A. et al. CNS disease diminishes the therapeutic functionality of bone marrow mesenchymal stem cells. Exp. Neurol. 295, 222–232 (2017).

TFRC (1:200, mouse, Thermo Fisher Scientific, 13-6800, clone H68.4): Vanlandewijck, M. et al. A molecular atlas of cell types and zonation in the brain vasculature. Nature 554, 475–480 (2018)

TFRC (1:200, rat, Novus Biologicals, NB100-64979, clone 8D3): Vanlandewijck, M. et al. A molecular atlas of cell types and zonation in the brain vasculature. Nature 554, 475–480 (2018)

Ki67 (1:1000, rat, eBioscience, 14-5698, clone SolA15): Magnusson, J. P. J. P. et al. A latent neurogenic program in astrocytes regulated by Notch signaling in the mouse. Science (80-.). 346, 237–41 (2014); https://www.thermofisher.com/antibody/product/Ki-67-Antibody-clone-SolA15-Monoclonal/14-5698-82

Vimentin (1:1000, chicken, Chemicon, AB5733): https://www.merckmillipore.com/SE/en/product/Anti-Vimentin-Antibody,MM_NF-AB5733?ReferrerURL=https%3A%2F%2Fwww.google.com%2F&bd=1; Barnabé-Heider, F. et al. Origin of new glial cells in intact and injured adult spinal cord. Cell Stem Cell 7, 470–482 (2010); Meletis, K. et al. Spinal cord injury reveals multilineage differentiation of ependymal cells. PLoS Biol. 6, 1494–1507 (2008).

Vimentin (1:500, rabbit, Abcam, ab92547, clone EPR3776): Xie, C. et al. Astrocytic YAP promotes the formation of glia scars and neural regeneration after spinal cord injury. J. Neurosci. 2229–19 (2020). doi:10.1523/JNEUROSCI.2229-19.2020; https://www.abcam.com/vimentin-antibody-epr3776-cytoskeleton-marker-ab92547.html#lb

Desmin (1:200, rabbit, Abcam, ab15200): Göritz, C. et al. A pericyte origin of spinal cord scar tissue. Science (80-.). 333, (2011); Vanlandewijck, M. et al. A molecular atlas of cell types and zonation in the brain vasculature. Nature 554, 475–480 (2018); Diéguez-Hurtado, R. et al. Loss of the transcription factor RBPJ induces disease-promoting properties in brain pericytes. Nat. Commun. 10, 2817 (2019)

αSMA (1:500, mouse directly conjugated to Cy3, Sigma-Aldrich, C6198, clone 1A4): Vanlandewijck, M. et al. A molecular atlas of cell types and zonation in the brain vasculature. Nature 554, 475–480 (2018); https://www.sigmaaldrich.com/SE/en/product/sigma/c6198?context=product

αSMA (1:100, rabbit, Abcam, ab5694): Assinck, P. et al. Myelinogenic plasticity of oligodendrocyte precursor cells following spinal cord contusion injury. J. Neurosci. 37, 8635–8654 (2017); Guimarães-Camboa, N. et al. Pericytes of Multiple Organs Do Not Behave as Mesenchymal Stem Cells In Vivo. Cell Stem Cell 20, 345-359.e5 (2017).

UEA-1 (1:200, directly conjugated to FITC or Rhodamine, Vector Labs, FL-1061 or RL-1062, respectively): Winkler, E. A. et al. Blood–spinal cord barrier breakdown and pericyte reductions in amyotrophic lateral sclerosis. Acta Neuropathol. 125, 111–120 (2013).

Lycopersicon Esculentum (Tomato) lectin (biotinylated, Vector Labs, B-1175-1): Robertson, R. T. et al. Use of labeled tomato lectin for imaging vasculature structures. Histochem. Cell Biol. 143, 225–234 (2015); https://vectorlabs.com/biotinylated-lycopersicon-esculentum-tomato-lectin-lel-tl.html

FluoroMyelin Red fluorescent myelin stain (1:500, Invitrogen, F34652): Dorrier, C. E. et al. CNS fibroblasts form a fibrotic scar in response to immune cell infiltration. Nat. Neurosci. 24, 234–244 (2021); https://www.thermofisher.com/order/catalog/product/F34652?SID=srch-hj-F34652#/F34652?SID=srch-hj-F34652

Control tissue sections were stained with secondary antibody alone.

Eukaryotic cell lines

Policy information about cell lines

Cell line source(s)	GL261 mouse glioma cell line (Leibniz Institute DSMZ-German Collection of Microorganisms and Cell Cultures GmbH; DSMZ, ACC 802).
Authentication	The cell line has been authenticated by the provider. Confirmed as mouse by species-PCR and COI barcoding; inbred strain analysis excluded DBA/s, Swiss, Balb/c, C3H and 129Sv.
Mycoplasma contamination	Cell line tested negative for Mycoplasma contamination.
Commonly misidentified lines (See ICLAC register)	No commonly misidentified cell lines have been used in the study.

Animals and other organisms

Policy information about studies involving animals; ARRIVE guidelines recommended for reporting animal research

Laboratory animals

The following adult (8 weeks of age or older) female and male mice were used in this study:

- GLAST-CreERT2 transgenic mice (Slezak, M. et al. Transgenic mice for conditional gene manipulation in astroglial cells. Glia 55, 1565-1576, doi:10.1002/glia.20570 (2007)) in a C57Bl/6J genetic background.

- Rosa26-enhanced yellow fluorescent protein (EYFP) Cre-reporter mice (obtained from the Jackson Laboratory, B6.129X1-Gt(Rosa)26Sortm1(EYFP)Cos/J, JAX stock: 006148) in a C57Bl/6J genetic background.

- Rosa26-tdTomato Cre-reporter mice (obtained from the Jackson Laboratory, B6.Cg-Gt(Rosa)26Sortm14(CAG-tdTomato)Hze/J, JAX stock: 007914) in a C57Bl/6J genetic background.

- HRas–/–;NRas–/–; KRas loxP/loxP (Rasless) mice (Drosten, M. et al. Genetic analysis of Ras signalling pathways in cell proliferation, migration and survival. EMBO J. 29, 1091–1104 (2010)) in a C57Bl/6J genetic background.

Wild animals	The study did not involve wild animals.
Field-collected samples	The study did not involve samples collected from the field.
Ethics oversight	Experimental procedures involving animals were performed in accordance with the Swedish and European Union guidelines and approved by the institutional ethical committees (Stockholms Norra Djurförsöksetiska Nämnd or Malmö/Lunds Djurförsöksetiska Nämnd).

Note that full information on the approval of the study protocol must also be provided in the manuscript.

Human research participants

Policy information about studies involving human research participants

Population characteristics	Brain and spinal cord tissue samples were obtained from international biobanks and collaborators and only available for distribution from a third party. More detailed information can be found in the Material and Methods section under "Human tissue collection and ethical compliance". Supplementary tables 1-4 show the clinical and neuropathological data of all subjects included in the study.<br><br>- Stroke post mortem and glioblastoma tissue samples:<br>For stroke samples, tissue collection was performed in such cases that had a history of a supratentorial territorial or lacunar stroke involving cerebral cortical and subcortical areas and who died from non-neurological cause. Healthy occipital cortex and healthy tissue from the contralateral hemisphere in corresponding topography served as control tissue.<br>For glioblastoma samples, initial histological analysis for verification of tumor type and WHO classification was performed by local experienced neuropathologists on formalin embedded tissue pieces. Standard hematoxylin and eosin staining, and immunohistochemical analyses for GFAP, MAP2 and pan cytokeratin-1 (KL-1) confirmed tumor types (glioblastoma, WHO grade IV).<br><br>- Multiple sclerosis post mortem tissue samples:<br>Multiple sclerosis tissue samples and associated clinical and neuropathological data were supplied by the UK Multiple Sclerosis Tissue Bank, supported by the Multiple Sclerosis Society of Great Britain and Northern Ireland, in partnership with Imperial College London. All tissue sample characterization and identification were done by independent neuropathologist following standard criteria. For healthy controls, tissue had no pathological conditions and diagnosis.<br><br>-Spinal cord injury post mortem tissue samples:<br>Human spinal cord injury samples and related clinical and neuropathologial information were obtained from the International Spinal Cord Injury Biobank (ISCIB), which is housed in Vancouver, BC, Canada.
Recruitment	All biospecimens were collected from consented participants or their next-of-kin for use in future research.
Ethics oversight	This study complied with all relevant ethical regulations regarding experiments involving human tissue samples. Ethical permission for this study was granted by the Regional Ethics Committee of Sweden (2010/313-31/3).<br><br>Stroke and glioblastoma samples:<br>The institutional review boards and the local ethics committee of the Medical Faculty of the University of Erlangen-Nürnberg, Erlangen, Germany, approved the study (issued ethical votes No. 4821, 104_1313 and 331_14B) and informed consent was obtained from the relatives of all analyzed patients.<br><br>- Multiple sclerosis samples:<br>The ethical permission was granted by the Regional Ethics Committee for UK (Research Ethics Committee for Wales, 08/MRE09/31+5). All samples have been donated with informed consent for use in future research.<br><br>-Spinal cord injury samples:<br>Permission for post-mortem spinal cord acquisition and for sharing of biospecimens was granted by the Clinical Research Ethics Board (CREB) of the University of British Columbia, Vancouver, Canada (Ethics certificate of full board approval H19-00690). All biospecimens were collected from consented participants or their next-of-kin.

Note that full information on the approval of the study protocol must also be provided in the manuscript.
